# Efficiency of platelet-rich plasma therapy in knee osteoarthritis does not depend on level of cartilage damage

**DOI:** 10.1186/s13018-019-1203-0

**Published:** 2019-05-24

**Authors:** Rene Burchard, Hanno Huflage, Christian Soost, Oliver Richter, Bertil Bouillon, Jan Adriaan Graw

**Affiliations:** 1 Department of Health, University of Witten/Herdecke, Witten, Germany; 2 Department of Trauma- and Orthopaedic Surgery, Kreisklinikum Siegen, Siegen, Germany; 3 School of Science and Technology, University of Siegen, Siegen, Germany; 4 Department of Statistics an Econometrics, University of Siegen, Siegen, Germany; 5 Institute of Diagnostic Radiology at Kreisklinikum Siegen, Siegen, Germany; 6 Department of Orthopaedics, Trauma Surgery and Sports Traumatology, Kliniken der Stadt Köln, Köln, Germany; 7 Department of Anaesthesiology and Operative Intensive Care Medicine (CCM, CVK) Charité, Universitätsmedizin Berlin, Berlin, Germany; 8 Berlin Institute of Health (BIH), Berlin, Germany

**Keywords:** Osteoarthritis, Knee, Platelet-rich plasma, Cartilage damage

## Abstract

**Objectives:**

Osteoarthritis of the knee is common and often leads to significant physical disability. While classic conservative therapeutic approaches aim for symptoms like pain and inflammation, procedures like the intraarticular application of hyaluronic acids (HA) or platelet-rich plasma (PRP) are thought to stimulate the endogenous HA production, stop catabolism of cartilage tissue, and promote tissue regeneration. To analyse whether the positive effects of PRP injections are associated with the level of cartilage damage, patient satisfaction with the treatment was correlated with the level of knee joint osteoarthritis quantified by MRI.

**Methods:**

PRP was performed with a low-leukocyte autologous conditioned plasma (ACP) system in 59 patients. A pre-treatment MRI was performed and a Whole-Organ MRI Score (WORMS) was used to score the level of knee osteoarthritis by 14 features: integrity of the cartilage, affection of the bone marrow, subcortical cysts, bone attrition, osteophytes, integrity of the menisci and ligaments, presence of synovitis, loose bodies, and periarticular cysts. A multivariate analysis with ordinary least squares regressions was used.

**Results:**

Although pain symptoms and severity of clinical osteoarthritis symptoms decreased, regression analysis could not detect a correlation between the degree of cartilage damage measured by the WORMS score and a positive response to PRP therapy.

**Conclusion:**

This study suggests that intraarticular injection of PRP might improve osteoarthritis symptoms and reduces the pain in patients suffering from osteoarthritis of the knee joint independent from the level of cartilage damages quantified by the whole-organ MRI scoring method WORMS.

## Introduction

Osteoarthritis of the knee has a prevalence of 10–18% and without treatment often leads to significant physical disability [1–3]. Conservative therapies to relieve pain and inactivity include treatment with nonsteroidal anti-inflammatory drugs (NSAIDs), physical activity, and braces. In addition, invasive therapeutic approaches include injections of steroids, hyaluronic acids (HA), or platelet-rich plasma (PRP) [4]. When conservative treatment approaches become insufficient, surgical treatment options include arthroscopic debridement and lavage, osteotomy, and unicompartimental or total knee arthroplasty [5]. However, surgical treatment of knee osteoarthritis should only be performed when conservative treatment options fail. This strategy is particularly relevant when cartilage damages are only mild to moderate [6].

Mechanical or biochemical non-physiological stimuli and a loss of bone-cartilage homeostasis were identified as triggering factors for the development of osteoarthritis [7]. While classic conservative methods like treatment with NSAIDs mainly target symptoms like pain and inflammation, alternative procedures like intraarticular application of HA or PRP to stimulate the endogenous HA production, stop catabolism of cartilage tissue and promote cellular metabolism and tissue regeneration [8, 9]. Many studies have compared the clinical outcome of these competitive techniques with scoring systems such as the visual analogue scale (VAS) to quantify the severity of pain or the Western Ontario and McMaster Universities Osteoarthritis Index (WOMAC) the most widely used assessment to evaluate pain, stiffness, and physical functions in arthritis research [8, 10–17]. Although results are inhomogeneous, PRP therapy appears to have a positive effect on biological cartilage repair [9, 15].

While conventional radiography provides high-resolution images of bone tissue, a direct visualisation of cartilage is not possible. In addition, bone marrow changes like bone marrow edema are not detected by conventional radiography. In contrast, magnetic resonance imaging (MRI) is more suitable for detailed investigations of osteoarthritic joints with mild to severe cartilage damages [18–20]. Because MRI can discriminate all articular tissues, the current study used MRI as the ideal suited diagnostic method to evaluate the cartilage status.

The main objective of this study was to analyse whether patient satisfaction with PRP therapy was associated with the degree of cartilage damage quantified with MRI.

## Methods

The Medical Ethics Committee of the Medical Council Westphalia-Lippe approved this study (number of ethical approval: 2015-685-f-S). Written informed consent was obtained from all study participants before participation.

All patients > 18 years with MRI-proven knee osteoarthritis, walking ability, and indication for PRP treatment were enrolled in the study at our centre in 2016 after informed consent was obtained. Patients with an age under 18 years, MRI-imaging without signs of knee osteoarthritis, limitation of motion range for flexion < 90° and extension > 20°, arthroscopic or open surgery within the past 3 months, fibromyalgia, chronic fatigue syndrome, coagulation disorder, thrombocytopenia (< 150.000 platelets per mm^3^), patients who received intra-articular injection of steroids, anaesthetics, or viscosupplementation within the last 12 months, pregnant patients and patients with chronic diseases, such as rheumatoid arthritis, or significant cardiovascular comorbidities, current infections, cancer, or diabetes, and patients with severe damage of the homolateral hip or ankle were excluded from this study. Patients were not treated with anti-inflammatory drugs during the period of PRP treatment. The Western Ontario and McMaster Universities Osteoarthritis Index (WOMAC) and the visual analogue scale (VAS) were surveyed before the first injection and after a mean follow-up of 24 weeks [16, 17].

To evaluate the level of osteoarthritis, a pre-treatment MRI was performed and analysed by a senior consultant radiologist with the whole-organ MRI scoring method (WORMS) according to Peterfy and colleagues [21]. The WORMS method scores the level of knee osteoarthritis by 14 features, such as integrity of the cartilage (cartilage damage), affection of the bone marrow, subcortical cysts, bone attrition, osteophytes, integrity of the menisci and ligaments, presence of synovitis, loose bodies, and periarticular cysts [21]. Furthermore, the cartilage damage was used for regression analysis as a dependent variable (WORMS Cartilage Score). A 1.5-T whole-body scanner with a circumferential knee coil (Philips Ingenia® 1.5 T, Philips GmbH, Hamburg, Germany) was used for imaging. Scanner settings and sequences were adjusted according to Peterfy and colleagues [21].

PRP was performed with a low-leukocyte autologous conditioned plasma (ACP) system by drawing 15 ml of blood with the ACP Double Syringe® (Arthrex, Naples, USA). Blood was centrifuged at 1500 rpm for 5 min in a Rotofix® 32 A centrifuge (Andreas Hettich GmbH, Tuttlingen, Germany). The ACP Double Syringe® provides a closed mechanism to separate the plasma from the cell pool. Five millilitres of leukocyte-low and platelet-rich plasma was extracted. The obtained plasma was injected into the knee joint within 5 min after extraction. Patients were treated once a week for three times.

### Statistics

A power analysis was performed with the software package G*Power® to compute the a priori required sample size for the mean comparisons (*n* = 32) and the regression analysis (*n* = 50) [22]. Data were analysed with statistical software package SPSS® Version 25 (IBM, Armonk, North Castle, New York, USA). The mean differences were analysed with the Wilcoxon signed-rank test. For the multivariate analysis, OLS (ordinary least squares) regressions were used. OLS regression (multiple linear regression model) is suitable for the exact quantification of causal relationships with metric independent variables. Data are presented as mean values with standard error of the mean (SEM). A *p* value of less than 0.05 was considered significant.

## Results

Fifty-nine patients (52.5% male) with a mean age of 58.78 ± 1.54 years and a body mass index (BMI) of 26.11 ± 0.50 underwent PRP therapy for osteoarthritis of the knee. According to the pre-treatment WORMS Cartilage Score, the severity of osteoarthritis was quantified as mild in 12 (20.3%), moderate in 33 (55.9%), and severe in 14 (23.7%) of the cases.

**Fig. 1:**
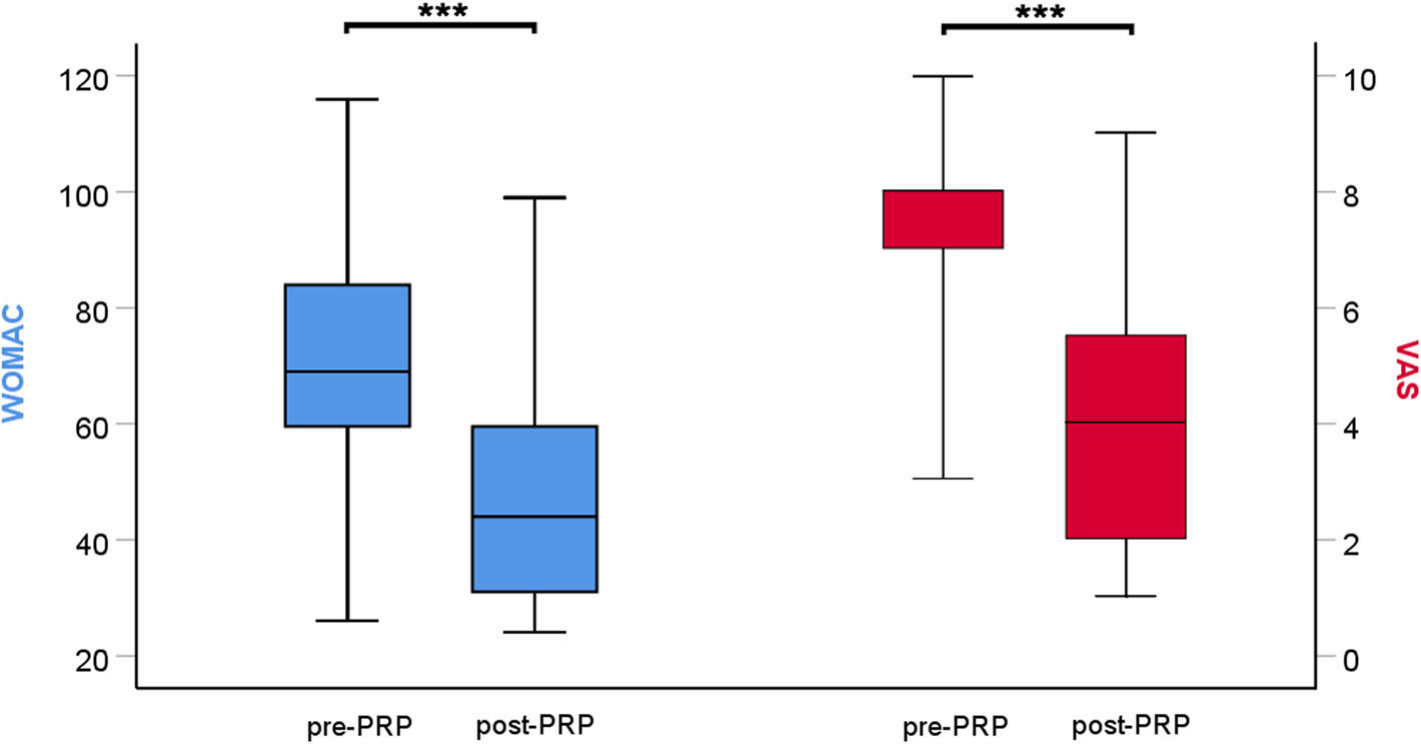
Change of VAS and WOMAC 24 weeks after PRP therapy. Absolute WOMAC results (blue) and VAS results (red) were compared pre PRP therapy and 24 weeks after PRP therapy

To analyse clinical outcome and patient satisfaction with PRP therapy, VAS and WOMAC scores were surveyed before the first injection and after a follow-up of 24.2 ± 0.1 weeks (Table 1). VAS decreased after PRP therapy by 3.58 ± 0.34 points (*p* <  0.001) and the WOMAC-Score decreased by 23.51 ± 2.82 points (*p* <  0.001, Fig. 1). Detailed changes of the WOMAC-Score were collected for pain − 5.41 ± 0.61 points (*p* <  0.001), stiffness − 1.76 ± 0.31 points (*p* <  0.001), and physical function − 16.34 ± 2.00 points (*p* <  0.001). No significant differences were found for the level of osteoarthritis.

**Table 1:** Post-treatment changes of pain, stiffness, and physical function

Variable	Mean difference	SE	*p* value
VAS	− 3.58	0.34	< 0.001
WOMAC	− 23.51	2.82	< 0.001
WOMAC pain	− 5.41	0.61	< 0.001
WOMAC stiffness	− 1.76	0.31	< 0.001
WOMAC physical function	− 16.34	2.00	< 0.001

Changes of the visual analogue scale (VAS) and Western Ontario and McMaster Universities Osteoarthritis Index (WOMAC) scores after treatment with platelet-rich plasma (PRP); mean difference describes the change of the mean score after therapy with PRP compared to the mean score before PRP therapy; statistical comparisons are made with non-parametric Wilcoxon signed-rank test; *SE* standard error

To quantify cartilage damage in the osteoarthritic knee joints, MRI imaging was performed. MRI-based quantification of cartilage damage by the WORMS score showed a mean selective cartilage damage of 3.00 ± 0.15 points. The overall WORMS score had a mean value of 86.66 ± 5.75 points. Details of this analysis are shown in Table 2 [21].

**Table 2:** MRI-based WORMS analysis

	Cartilage	Bone marrow lesion	Subarticular cysts	Subarticular bone attrition	Osteophytes
LP	3.21 (*r* 0–6; SE 0.19)	0.29 (*r* 0–2; SE 0.08)	0.05 (*r* 0–1; SE 0.03)	0.41 (*r* 0–3; SE 0.10)	1.08 (*r* 0–5; SE 0.17)
MP	3.37 (*r* 0–6; SE 0.18)	0.32 (*r* 0–2; SE 0.07)	0.03 (*r* 0–1; SE 0.02)	0.49 (*r* 0–3; SE 0.09)	1.29 (*r* 0–6; SE 0.17)
MFa	3.26 (*r* 0–6; SE 0.21)	0.42 (*r* 0–2; SE 0.08)	0.15 (*r* 0–2; SE 0.05)	0.73 (*r* 0–3; SE 0.11)	1.68 (*r* 0–5; SE 0.18)
MFc	3.68 (*r* 0–6; SE 0.20)	0.61 (*r* 0–3; SE 0.10)	0.15 (*r* 0–2; SE 0.06)	0.73 (*r* 0–3; SE 0.12)	1.53 (*r* 0–5; SE 0.18)
MFp	2.77 (*r* 0–6; SE 0.24)	0.27 (*r* 0–2; SE 0.07)	0.37 (*r* 0–3; SE 0.10)	0.37 (*r* 0–2; SE 0.08)	2.27 (*r* 0–6; SE 0.24)
MTa	2.64 (*r* 0–6; SE 0.21)	0.22 (*r* 0–2; SE 0.06)	0.02 (*r* 0–1; SE 0.02)	0.31 (*r* 0–2; SE 0.08)	1.63 (*r* 0–4; SE 0.17)
MTc	3.52 (*r* 0–6; SE 0.20)	0.51 (*r* 0–3; SE 0.10)	0.12 (*r* 0–1; SE 0.04)	0.95 (*r* 0–3; SE 0.12)	1.41 (*r* 0–5; SE 0.17)
MTp	2.61 (*r* 0–5; SE 0.19)	0.32 (*r* 0–2; SE 0.08)	0.25 (*r* 0–2; SE 0.07)	0.41 (*r* 0–2; SE 0.08)	2.19 (*r* 0–5; SE 0.22)
LFa	3.06 (*r* 0–6; SE 0.22)	0.36 (*r* 0–3; SE 0.09)	0.15 (*r* 0–2; SE 0.05)	0.59 (*r* 0–2; SE 0.10)	1.32 (*r* 0–5; SE 0.17)
LFc	3.07 (*r* 0–6; SE 0.17)	0.29 (*r* 0–3; SE 0.09)	0.03 (*r* 0–1; SE 0.02)	0.41 (*r* 0–2; SE 0.10)	1.02 (*r* 0–4; SE 0.15)
LFp	2.32 (*r* 0–5; SE 0.22)	0.10 (*r* 0–2; SE 0.05)	0.03 (*r* 0–1; SE 0.02)	0.31 (*r* 0–2; SE 0.07)	1.37 (*r* 0–4; SE 0.18)
LTa	2.96 (*r* 0–6; SE 0.19)	0.17 (*r* 0–2; SE 0.07)	0.08 (*r* 0–2; SE 0.04)	0.39 (*r* 0–2; SE 0.08)	1.05 (*r* 0–6; SE 0.16)
LTc	3.04 (*r* 0–6; SE 0.22)	0.42 (*r* 0–3; SE 0.11)	0.12 (*r* 0–2; SE 0.05)	0.56 (*r* 0–3; SE 0.11)	1.27 (*r* 0–5; SE 0.17)
LTp	2.55 (*r* 0–6; SE 0.20)	0.29 (*r* 0–2; SE 0.08)	0.10 (*r* 0–2; SE 0.05)	0.29 (*r* 0–2; SE 0.08)	1.31 (*r* 0–5; SE 0.20)
S		0.17 (*r* 0–1; SE 0.05)	0.15 (*r* 0–2; SE 0.05)		
	Ligaments	Menisci	Synovitis	Overall	
	0.02 (*r* 0–1; SE 0.01)	1.00 (*r* 0–12; SE 0.14)	0.41 (*r* 0–2; SE 0.07)	78.65 (*r* 13–196; SE 5.60)	

Detailed results of the magnetic resonance imaging (MRI)-based Whole-Organ MRI Score. The analysis includes common MRI-findings for osteoarthritis in 15 anatomical sections of the knee joint; *LP* lateral patella, *MP* medial patella, *MF* medial femur, *MT* medial tibia, *LF* lateral femur, *LT* lateral tibia, *a* anterior, *c* central, *p* posterior, *S* subspinous

To examine whether a positive response to PRP therapy was associated with the degree of cartilage damage or osteoarthritis, regression analysis was performed. There was no correlation between the degree of osteoarthritis (overall WORMS score; *p* = 0.647) or the isolated degree of cartilage damage (WORMS Cartilage Score; *p* = 0.805) and a positive response to PRP therapy (Table 3). However, according to the measured VAS and WOMAC scores, female participants showed a greater WOMAC-Score reduction of osteoarthritis after PRP therapy compared to male participants (*p* = 0.004 depending on overall WORMS score and *p* = 0.003 depending on WORMS Cartilage Score) (Table 3). In addition, a Bravais-Pearson pair-wise correlation was performed to check the validity of the regression model (Table 4).

**Table 3:** Post-treatment changes of pain and activity level—regression analysis

Dependent variable	Change of VAS depending on WORMS overall	Change of VAS depending on WORMS Cartilage	Change of WOMAC depending on WORMS overall	Change of WOMAC depending on WORMS Cartilage
Constant	− 2.036 (.464)	− 1.900 (.498)	1.012 (.963)	− .224 (.992)
WORMS	.007 (.410)		− .032 (.647)	
WORMS Cartilage		.026 (.940)		.659 (.805)
Sex	− .895 (.204)	− 1.004 (.157)	16.442** (.004)	17.235** (.003)
BMI	− .018 (.838)	− .025 (.780)	.670 (.343)	.709 (.314)
Age	− .021 (.520)	− .011 (.753)	− .004 (.989)	− .084 (.747)
*n*	59	59	59	59

Regression analysis of post-treatment changes for pain and activity level with regard to MRI-proven cartilage damage level and common epidemiological data. Dependent variables are Change of VAS (visual analogue scale) and Change of WOMAC (Western Ontario and McMaster Universities Osteoarthritis Index). OLS regression coefficients with *p* values in parentheses (*p ≤ .05, **p ≤ .01, ***p ≤ .001 two-tailed)

**Table 4:** Post-treatment changes of pain and activity level—descriptive statistics

		Mean	SD	1	2	3	4	5	6	7
1	Change of VAS	− 3.58	2.59	1						
2	Change of WOMAC	23.51	21.66	− .780**	1					
3	WORMS	81.66	44.15	.118	− .142	1				
4	WORMS Cartilage	3.00	1.17	.024	− .057	.951*	1			
5	Sex	.47	.50	− .197	.393**	− .190	− .182	1		
6	BMI	26.12	3.88	− .041	.117	− .031	.017	− .012	1	
7	Age	58.78	11.82	− .046	− .017	.417**	.463**	− .008	.125	1

Descriptive statistics of post-treatment changes of pain and activity level depending on the MRI-proven cartilage damage level and common epidemiological data; *SD* standard deviation and *p ≤ .05, **p ≤ .01 (two-tailed)

## Discussion

To analyse whether the positive effects of PRP injections are associated with the level of cartilage damage, the satisfaction of the patients with their treatment was correlated with the level of knee joint osteoarthritis quantified by MRI. Regression analysis could not detect a correlation between the level of cartilage damage or the level of osteoarthritis and a positive response to PRP therapy in the studied patient population.

A fair number of studies with different injection treatments for patients suffering from osteoarthritis of the knee have been completed so far [8, 10–15]. For mild to moderate cartilage damages, Filardo and colleagues found a similar clinical outcome after PRP therapy compared to therapy with HA [8]. However, they only used radiographic images to determine the degenerative changes (Kellgren-Lawrence-Score 1–3) and excluded cases with severe osteoarthritis. Similar results were reported by other study groups [10–12]. Frequently, an advantage of PRP therapy compared to HA application was seen for the treatment of mild-moderate knee osteoarthritis in younger patients and in male patients [13–15, 23–25]. Nevertheless, all of these studies either used a different PRP preparation protocol or did not provide detailed information to reproduce the treatment algorithm [26]. For depletion of leukocytes, Milants and colleagues suggested a method with only one spinning cycle during the centrifugation step for preparation of the PRP [27]. Moreover, they reported a platelet concentration of fivefold lower than the baseline [27]. For the current study, the Arthrex® ACP protocol according to Marlovits and colleagues was used [28].

Our results suggest that the level of cartilage damage after PRP therapy in knee osteoarthritis is not associated with the clinical outcome measured by the VAS and WOMAC scoring systems. Similar to findings of Jubert and colleagues, our data suggest that late-stage osteoarthritis does not seem to be an exclusion criterion for PRP therapy because clinical outcome did not differ between patients with mild to moderate and severe disease stages [29]. While restorative effects on the cartilage by PRP therapy have been discussed controversially, anti-inflammatory effects, down-regulation of cytokine levels, and joint homeostasis might explain favourable effects in patients with severe osteoarthritis [30, 31]. However, currently, there is a lack of evidence to support the theory of a regeneration of substantial or irreversible cartilage damages by PRP therapy [32]. Furthermore, like other study groups, we could not observe a superior effect of PRP therapy when treating younger men [11, 33].

Most of the above-mentioned studies compared results of PRP therapy to the disease progress of osteoarthritis using weight-bearing radiography to stage the cartilage damage level according to the Kellgren-Lawrence score, which is based on radiographic images exclusively [8]. However, detectable radiographic changes appear mainly in the later stages of the disease [19]. MRI technology provides robust acquisition protocols to study progress and level of osteoarthritis in the knee [20]. Therefore, in the current study, the complex and high detailed MRI-based WORMS protocol was used to stage the level of cartilage damages [21].

Besides the strengths of this study such as the use of detailed examination techniques with MRI and inclusion of patients with severe osteoarthritis stages, this study has several limitations. Besides the single-centre design and the low number of cases, the lack of a placebo control group, and a rather short-term follow-up limit the conclusions. Furthermore, the population in the presented study is older compared to other investigations in the literature [34–36].

## Conclusion

The findings of the current study suggest that positive effects of intraarticular injections of PRP might improve quality of life and reduce the pain of patients suffering from osteoarthritis of the knee joint independent from the level of cartilage damage.

## Data Availability

Data are available via the corresponding author.

## Abbreviations

ACP: Autologous conditioned plasma
MRI: Magnetic resonance imaging
PRP: Platelet-rich plasma
VAS: Visual analogue scale
WOMAC: Western Ontario and McMaster Universities Osteoarthritis Index
WORMS: Whole-organ MRI scoring method
